# Differences in the Development of Internal Reproductive Organs, Feeding Amount and Nutrient Storage between Pre-Diapause and Pre-Reproductive *Harmonia axyridis* Adults

**DOI:** 10.3390/insects10080243

**Published:** 2019-08-06

**Authors:** Qiao Gao, Bing-Xin Wei, Wen Liu, Jia-Lu Wang, Xing-Miao Zhou, Xiao-Ping Wang

**Affiliations:** Hubei Key Laboratory of Insect Resources Utilization and Sustainable Pest Management, College of Plant Science and Technology, Huazhong Agricultural University, Wuhan 430070, China

**Keywords:** *Harmonia axyridis*, diapause, photoperiod, internal reproductive organs, feeding amount, nutrient storage

## Abstract

Diapause control is one of the successful methods for long-term cold storage of biological control organisms without decreasing their fitness. Sufficient preparation before diapause is essential for successful diapause initiation and maintenance. *Harmonia axyridis*, an important biocontrol agent in its native place, can enter reproductive diapause for overwintering. However, the key preparatory events before diapause in *H. axyridis*, such as specific developmental trajectory, timing, and physiological changes, remain unclear. We compared differences in the development of internal reproductive organs, feeding amount and nutrient storage between pre-diapause and pre-reproductive adult *H. axyridis* which had been reared at 20 °C under a short-day (10L:14D) and long-day (14L:10D) condition, respectively. The results showed that there were obvious morphological differences in internal reproductive organs of diapause and reproductive *H. axyridis*. The development of internal reproductive organs was suppressed at early adult stage in pre-diapause beetles compared to pre-reproductive beetles. Feeding amount in both pre-diapause and pre-reproductive beetles increased for more than ten days after eclosion. The feeding amount of pre-diapause beetles sharply decreased from the 15th day after eclosion in females and the 14th day after eclosion in males, which implied the initiation of diapause. During the pre-diapause stage, carbohydrates and lipids were mainly accumulated by females, whereas males mainly accumulated carbohydrates. Our results not only provide basic information about the diapause process of pre-diapause stage, but also give tips to better utilization of diapause strategy during mass production and storage of *H. axyridis*.

## 1. Introduction

The multicolored Asian lady beetle *Harmonia axyridis* is a well-known biological control agent for aphids and coccids in its native places, such as Japan and China [1,2,3,4]. Although *H. axyridis* is considered as an invasive species in Europe and North America [5,6,7,8], it is commercially reared and widely used as a biocontrol agent in its native places, due to its strong ability to control aphids and its environmental adaptation [4,9]. One of the most important environmental adaptation strategies for *H. axyridis* is reproductive diapause. *H. axyridis* enters a long-term reproductive diapause phase to avoid adverse environmental conditions and maintain population during the overwintering process [10,11,12,13]. The mechanism used by diapause insects to survive low temperatures provides new ideas for cold storage of commercially viable biological control organisms [14]. Long-term cold storage of some parasitoids, such as *Trissolcus basalis*, *Telenomus podisi* and *Habrobracon hebetor*, has been successfully achieved by inducing diapause, without decreasing their fitness [15,16,17,18]. Recent research showed that field-collected overwintering *H. axyridis* can be stored at 3 °C for 150 days with no subsequent loss of fitness [18], which implies the possibility of long-term storage of commercial mass reared *H. axyridis* by inducing diapause. However, the diapause characteristics of *H. axyridis* remain largely unknown. A better understanding of the diapause strategy of *H. axyridis* could help us improve the mass rearing and storage of the species for biological control.

Previous studies have indicated that *H. axyridis* is a temperate insect with facultative winter diapause, and that both sexes can enter diapause under short-day (SD) conditions [10,19,20,21]. Diapause beetles are characterized by decreased respiration, an undeveloped ovary, and a well-developed fat body [10,22]. Before *H. axyridis* enters diapause, it undergoes diapause induction and preparation stage [23]. The early adult stage of *H. axyridis* is the most sensitive stage to photoperiod and the photoperiod experienced by the pre-imaginal stage has no significant effect on the diapause induction [22]. More than 90% of beetles entered diapause when the newly emerged adults reared at 20 °C under a SD (10L:14D) condition for 20 days, whereas those reared under a long-day (LD) (14L:10D) condition at the same temperature became reproductive [22]. Although the environmental induction of diapause in *H. axyridis* has been well documented, little is known about the specific trajectory of reproductive development, timing, and physiological changes that occur during the pre-diapause stage.

Insects often undergo arrested development during the pre-diapause preparatory stage [24,25,26], along with the accumulation of lipids, carbohydrates, and proteins [27,28]. Insufficient diapause preparation can cause insects to fail to enter diapause, advance the termination of diapause, or die during diapause [27,29]. In order to uncover the pre-diapause preparatory events in *H. axyridis*, we compared the internal reproductive organs, developmental trajectories of the ovary, vas deferens, ejaculatory duct and male accessory glands, and daily feeding amount and nutrient storage, between pre-diapause and pre-reproductive adults.

## 2. Materials and Methods

### 2.1. Insects

More than 100 female and male *H. axyridis* of the Red-nSpots color pattern [30], which is the dominant local population [31], were collected in Wuhan, China, May, 2018 (30°28′ N 114°21′ E). Offspring of these insects were reared in the laboratory at 25 °C, under a 14L:10D photoperiod and fed on pea aphids, *Acyrthosiphon pisum*. Two hundred larvae were reared in a 40 × 40 × 40 cm cage containing broad bean *Vicia faba* seedlings infected with pea aphids. Foods were always offered ad libitum. Photoperiod treatments were initiated after pupation, as the pre-imaginal stage is insensitive to photoperiod [22]. Fresh pupae were moved to an incubator (HP-250-GS, Wuhan Ruihua Instrument and Equipment, Wuhan, China) and randomly assigned two different photoperiod treatments: an LD condition (14L:10D) to induce reproductive development and an SD condition (10L:14D) to induce diapause. Pupae in both treatments were kept at 20 °C. Females and males in both treatments were segregated immediately after eclosion. Adults of the same sex were reared in transparent round boxes (diameter 10 cm, height 5 cm; 20 adults per box). Fresh pea aphids were offered ad libitum and replenished daily. Female and male beetles reared at 20 °C under a SD condition enter diapause after feeding for 20 days, whereas those reared at 20 °C under a LD photoperiod become reproductive [22]. The beetles used in the following experiments were treated with photoperiod and rearing as described above.

### 2.2. Experimental Procedures

#### 2.2.1. Dissecting and Photographing Internal Reproductive Organs

Female and male beetles reared at 20 °C under a SD condition enter diapause after feeding for 20 days, whereas those reared at 20 °C under a LD photoperiod become reproductive. The internal reproductive organs of unmated diapause and reproductive beetles were dissected in a wax dish containing phosphate buffer solution (PBS) and photographed with a digital camera (Nikon D5100, Nikon Imaging (China) Sales, Wuhan, China) mounted on a stereo microscope (SMZ-t4, Chong Qing Optec Instrument, Chongqing, China).

#### 2.2.2. Developmental Status of Ovary, Vas Deferens, Ejaculatory Duct and Accessory Gland

The internal reproductive organs of 10–15 beetles reared under LD and SD condition for 0, 2, 4, 6, 8, 10, 12, 14, 16, 18 and 20 days were dissected and photographed as described above. The developmental status of the ovary was distinguished into five stages according to Sakurai et al. (1986) [32]. The diameter of the vas deferens, ejaculatory duct, and the basal accessory gland were measured using ScopePhoto 3.0.

#### 2.2.3. Changes in Daily Feeding Amount

The daily feeding amount of 15 female and male beetles from each treatment group was evaluated for 20 days using the method described by Soares et al. (2003) [33]. Beetles from each treatment group were reared individually in Petri dishes (diameter: 5 cm, height: 2.5 cm). Fifty similar sized adult pea aphids were provided daily, and the number of surviving aphids was counted after 24 h. Aphids were replenished after each daily count. Petri dishes containing just 50 aphids without beetles served as the control. Mortality in the control was low. Daily feeding amount was calculated according to the following model (Soares et al. 2003) [33]:

$$V_{0}=\left(A-a_{24}\right)\times ra_{24}$$

where *V*_0_ refers to number of aphids eaten, *A* refers to number of aphids available, *a*_24_ refers to number of aphids alive after 24 h, and *ra*_24_ refers to ratio of aphids found alive after 24 h in the control treatment.

#### 2.2.4. Determination of Nutrient Content

Nutrient storage is a consistent and significant preparatory event of diapause insects [27]. To clarify differences in nutrient storage between pre-diapause and pre-reproductive *H. axyridis*, we measured the protein, carbohydrate, and lipid content of female and male beetles from each photoperiod treatment group at 20 °C.

*Sample collection.* Beetles from each treatment group were sampled at 0, 2, 4, 6, 8, 10, 12, 14, 16, 18 and 20 days after eclosion. We set 5 biological replicates for carbohydrates and protein content measurement, and 4 beetles were used for each biological replicate. Beetles from each sampling time point of the two treatment groups were collected to measure the fresh weight, and then were frozen in liquid nitrogen and stored at −80 °C for further measurement. We set 10 biological replicates for lipid content measurement, and 1 beetle was used for each biological replicate. Beetles from each sampling time point of the two treatment groups was collected, and quickly frozen in liquid nitrogen and stored at −80 °C for lipid content measurement.

*Protein content.* Protein content was measured using the bicinchoninic acid (BCA) system [34] according to the manufacturer’s protocol (Jiancheng Bioengineering Institute, Nanjing, China, Code A045-3). Briefly, samples were weighted and homogenized with precooling PBS (1 g insect/9 mL of PBS). Then, 1 mL of the homogenate was transferred into new tubes and centrifuged at 2500 rpm at 4 °C. The supernatant was harvested and measured according to the manufacturer’s protocol. Absorbance was measured with a microplate reader (Bio-Rad, Xmark, Berkeley, CA, USA) under 450 nm. Protein content was then calculated according to the manufacturer’s protocol.

*Carbohydrate content.* Carbohydrate content was measured using the anthrone-sulfuric acid method following the method described by Dubois et al. (1956) [35]. Briefly, samples were weighted and homogenized with precooling PBS (1 g insect/9 mL of PBS) and 400 µL 10% trichloroacetic acid. Then, 1 mL of the homogenate was transferred into new tubes and centrifuged at 5000 rpm at 4 °C. The supernatant was harvested and diluted 10 times with PBS. 250 µL diluted solutions were then mixed with 1000 µL 0.2% anthrone-sulfuric acid in glass tubes, the tubes were placed in a 100 °C water bath for 10 min followed by a 20 min ice bath. The reaction solutions were then added to microplates and the absorbance measured with a microplate reader (Bio-Rad, Xmark, Berkeley, CA, USA) under 628 nm. The carbohydrate content was calculated from a standard curve adjusted for the dilution ratio.

*Lipid content.* Lipid content was measured using the method described by Folch et al. (1957) [36]. In brief, samples were dried for 24 h at 100 °C and their initial dry weight measured on an electronic balance (OHAUS AR2140, Shanghai, China). Lipids were then extracted using a chloroform and methanol (2:1) solution for 24 h at room temperature. After further extraction with a new chloroform and methanol solution for 6 h at room temperature, the dry weight was re-measured. The difference between the initial dry weight and the dry weight after lipid extraction was regarded as the weight of the stored lipids. Lipid content was calculated as the percentage of the weight of stored lipids to the initial dry weight.

#### 2.2.5. Statistical Analysis

Statistical analyses were conducted in SPSS 19 (IBM, Armonk, NY, USA). Levene’s tests were used to confirm homogeneity of variance and nonparametric tests (Kolmogorov-Smirnov test, K-S test) to confirm normality. Independent-Samples *t*-tests were used to assess the significance of differences in the size of internal reproductive organs, feeding amount and nutrient storage, between treatment groups (* *p* < 0.05, ** *p* < 0.01).

## 3. Results

### 3.1. Differences in the Development of Internal Reproductive Organs between Pre-Diapause and Pre-Reproductive Adult H. axyridis

#### 3.1.1. Morphological Differences in the Internal Reproductive Organs of Diapause and Reproductive Adult *H. axyridis*

**Figure 1:**
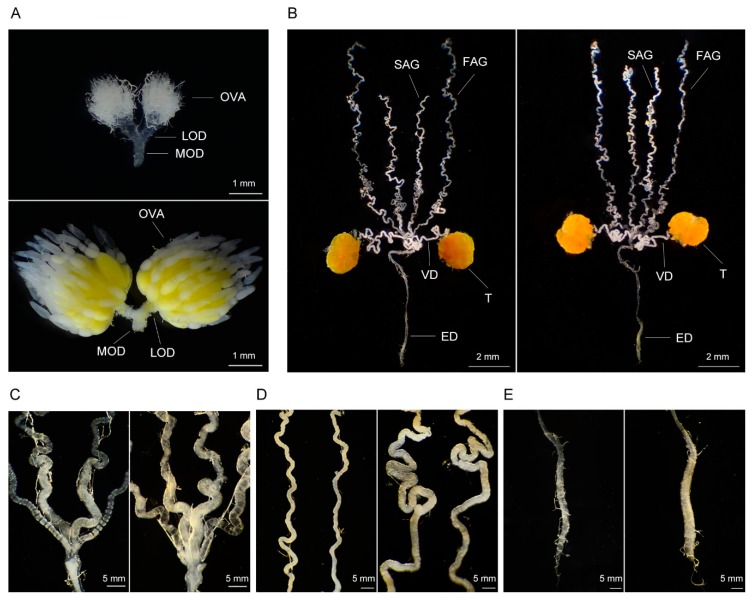
Morphological differences in the internal reproductive organs of diapause and reproductive adult *H. axyridis*. (**A**) Ovary of diapause (upper) and reproductive (lower) beetles; (**B**) male internal reproductive organs of diapause (left) and reproductive (right) beetles; (**C**) accessory gland of diapause (left) and reproductive (right) beetles; (**D**) vas deferens of diapause (left) and reproductive (right) beetles; (**E**) ejaculatory duct of diapause (left) and reproductive (right) beetles. OVA: Ovariole, LOD: Lateral oviduct, MOD: Median oviduct, FAG: First accessory gland, SAG: Second accessory gland, VD: Vas deferens, T: Testis, ED: Ejaculatory duct.

The arrested development of internal reproductive organs is one of the most remarkable features of diapause insects [24,27]. The internal reproductive organs of diapause beetles were suppressed compared to those of reproductive beetles (Figure 1A,B). The ovary of reproductive beetles was well developed, the ovarioles were markedly swollen, and each ovariole had three visible egg chambers filled with yellow yolk, the basal chamber of which contained mature eggs (Figure 1A). In contrast, the ovary of diapause beetles was infertile and the ovarioles were filiform with no recognizable egg chambers or yolk in the ovarioles (Figure 1A). The size of the vas deferens, ejaculatory duct and accessory gland of diapause beetles were also visibly smaller than those of reproductive beetles (Figure 1B–E).

#### 3.1.2. Differences in the Developmental Trajectory of Internal Reproductive Organs between Pre-Diapause and Pre-Reproductive Adult *H. axyridis*

**Figure 2:**
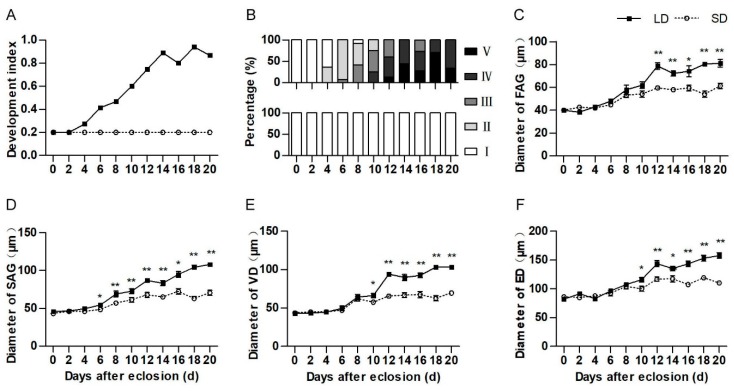
Differences in the development of internal reproductive organs in pre-diapause and pre-reproductive adult *H. axyridis*. (**A**) Developmental trajectory of the ovary in pre-reproductive (LD) beetles reared under a long-day condition and pre-diapause (SD) beetles reared under a short day condition, (**B**) changes in ovarian development during different developmental stages (I–V) in pre-diapause (upper) and pre-reproductive (lower) beetles, (**C**–**F**) developmental trajectory of the first accessory gland (FAG), second accessory gland (SAG), vas deferens (VD), and ejaculatory duct (ED), in pre-reproductive (LD) and pre-diapause (SD) beetles. All values are means ± standard error. Values in (A) and (B) were estimated from 15 independent biological replicates. Values in (**C**–**F**) were estimated from 10 independent biological replicates. Asterisks indicate significant differences determined by an independent samples *t*-test (* *p* < 0.05, ** *p* < 0.01).

We monitored and compared the developmental trajectory of the internal reproductive organs of pre-diapause and pre-reproductive *H. axyridis*. The ovary of pre-reproductive females developed to maturity (5th stage), while those of pre-diapause females remained immature (1st stage) (Figure 2A,B). Obvious differences in the appearance of the internal reproductive organs of pre-diapause and pre-reproductive female *H. axyridis* were apparent from 4 days after eclosion, whereas in males the differences became apparent after 6 days. The diameter of the male internal organs increased with age in both pre-diapause and pre-reproductive beetles (Figure 2C–F). The diameter of the first vas deferens, second vas deferens, ejaculatory duct, and accessory gland in pre-diapause males became significantly smaller than in pre-reproductive males after 12, 6 and 10 days after eclosion, respectively (first vas deferens: t = −6.225, df = 18, *p* < 0.001, second vas deferens: t = −2.215, df = 18, *p* = 0.042, ejaculatory duct: t = −2.642, df = 18, *p* = 0.017, and accessory gland: t = −2.643, df = 18, *p* = 0.017) (Figure 2C–F).

### 3.2. Differences in Feeding Amount between Pre-Diapause and Pre-Reproductive Adult H. axyridis

**Figure 3:**
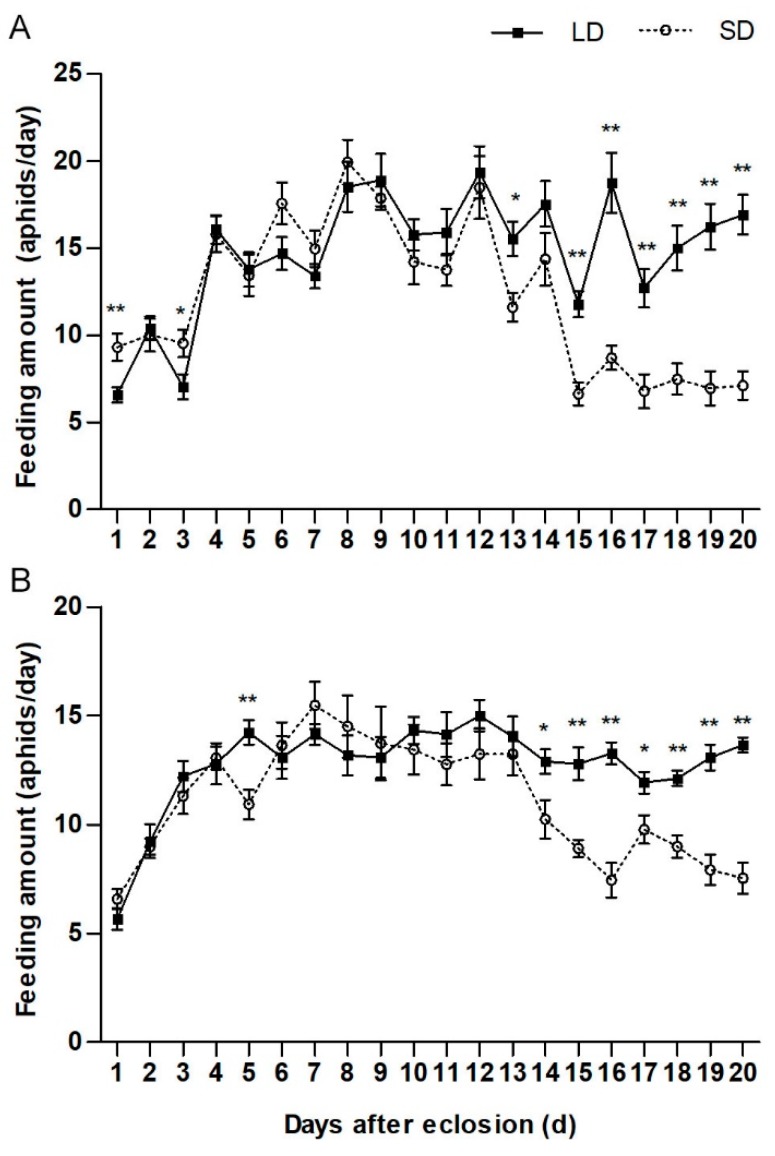
Dynamic changes in feeding amount in pre-diapause and pre-reproductive adult *H. axyridis*. Dynamic change in daily feeding amount of female (**A**) and male (**B**) *H. axyridis*. All values are means ± standard error estimated from 15 independent biological replicates. LD = pre-reproductive beetles reared under a long-day condition and SD = pre-diapause beetles reared under a short-day condition. Asterisks indicate significant differences identified by an independent samples *t*-test (* *p* < 0.05, ** *p* < 0.01).

Changes in daily feeding amount in pre-diapause and pre-reproductive beetles were recorded at 20 °C. The daily feeding amount of both pre-diapause and pre-reproductive beetles initially increased and did not significantly differ for more than ten days (Figure 3). The food consumption of female pre-diapause beetles sharply decreased from the 15th day after eclosion (Figure 3A), whereas that of pre-diapause males decreased from the 14th day after eclosion (Figure 3B) (the 15th day: t = −5.131, df = 28, *p* < 0.001, the 14th day: t = −2.534, df = 28, *p* = 0.021). Feeding amount of pre-diapause beetles then remained relatively low level, significantly lower than that of pre-reproductive beetles (Figure 3).

### 3.3. Differences in Nutrient Storage between Pre-Diapause and Pre-Reproductive Adult H. axyridis

**Figure 4:**
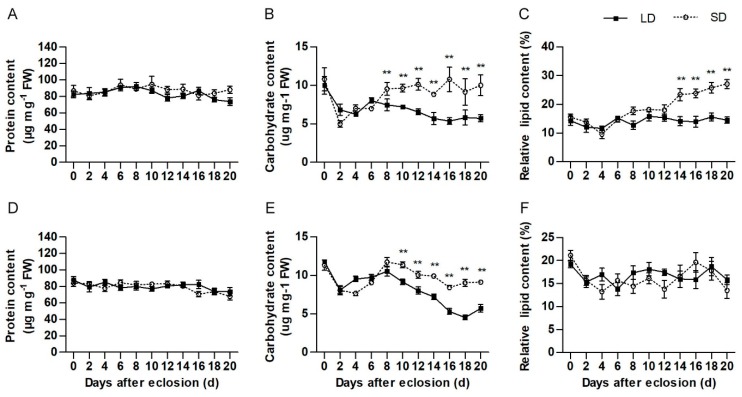
Differences in nutrient storage between pre-diapause and pre-reproductive adult *H. axyridis*. Protein accumulation in females (**A**) and males (**D**), Carbohydrate accumulation in females (**B**) and males (**E**), and lipid accumulation of females (**C**) and males (**F**). Values of protein and carbohydrate content are means ± standard error estimated from 5 independent biological replicates. Values of lipid content are means ± standard error estimated from 10 independent biological replicates. LD = pre-reproductive beetles reared under a long-day condition and SD = pre-diapause beetles reared under a short-day condition. Asterisks indicate significant differences determined by an independent samples *t*-test (* *p* < 0.05, ** *p* < 0.01).

The results indicate that both female and male pre-diapause beetles accumulated nutrients, and that these had accumulated more nutrients than pre-reproductive beetles after the 8th day after eclosion (Figure 4). Female pre-diapause beetles accumulated nutrients by 20 days after eclosion and had accumulated approximately 1.6 times more carbohydrates and 1.87 times more lipids than pre-reproductive female beetles (carbohydrates: t = 3.006, df = 8, *p* = 0.024; lipids: t = 6.556, df = 18, *p* < 0.001). Pre-diapause male beetles only accumulated carbohydrates, and by 20 days after eclosion these had accumulated approximately 1.75 times more carbohydrates than pre-reproductive males (t = 6.125, df = 8, *p* < 0.001). There was no significant difference in the protein content of pre-diapause and pre-reproductive beetles of both sex (Figure 4A,D).

## 4. Discussion

Better understanding the diapause strategy of biological control insects could help the exploit of its commercial mass production based on diapause control [14]. However, key events during the pre-diapause stage in *H. axyridis* remain unclear. Our results showed that at the condition of 20 °C combined with SD condition, the first 15 and 14 days after eclosion could be considered as pre-diapause stage for female and male adults, respectively. The pre-diapause stage of *H. axyridis* was characterized by suppressed internal reproductive organs, a continuous increase in feeding amount, and the accumulation of lipids and carbohydrates. These results clarify the key period and events of the pre-diapause stage, and provide a basis for further utilization of diapause strategy in the commercial mass rearing and long-term storage of *H. axyridis* for use as a biological control.

Arrested development of the internal reproductive organs is one of the most remarkable characteristics of reproductive diapause [24,27]. We found obvious morphological differences in the ovary, vas deferens, ejaculatory duct, and accessory gland of pre-diapause and pre-reproductive *H. axyridis*. These differences were clearly visible and can be used as diagnostic indicators of diapause in future research. In addition, the differences were apparent relatively early, on the 4th day after eclosion for females and on the 6th day for males, which implies that photoperiod affect the diapause and reproductive decision at the early adult stage [22].

Sufficient nutrient storage before diapause is not only essential for diapause initiation and maintenance, but also for post-diapause fitness [24,28,37]. For example, sufficient food is necessary for both the boll weevil *Anthonomus grandis grandis* and two-spotted spider mite *Tetranychus urticae* Koch to enter diapause [38,39]. Bark beetles *Ips typographus,* with less nutrient reserves, have higher mortality and lower post-diapause fitness [40]. We found that lipids and carbohydrates were significantly accumulated in pre-diapause beetles. Interestingly, there were sexual differences in the nutrient storage of pre-diapause beetles; females accumulated carbohydrates and lipids, while male beetles accumulated carbohydrates only. These differences may be due to the different post-diapause activities of females and males. Lipids are important for ovarian development [27,41], whereas carbohydrates provide most of the energy required for insect activity [27]. After diapause termination, male beetles become capable of mating as soon as females become receptive but have no further role to play in reproduction after mating is completed. Females, on the other hand, may need more energy reserves for egg maturation, and to search for suitable oviposition sites [24].

Insects increase their food intake prior to diapause to accumulate sufficient nutrient reserves to complete diapause successfully [27]. We found that the feeding amount of *H. axyridis* increased after eclosion and remained high for more than ten days after eclosion, with no significant difference in food amount between pre-diapause and pre-reproductive individuals during that period. There was a sharp decrease in the feeding amount of females from 15 days after eclosion, and in males, from 14 days after eclosion. This abrupt decrease in food intake and arrested development of the reproductive organs is a consistent feature of reproductive diapause insects [27,28,42,43]. The sharp decrease in feeding amount probably indicates the start of diapause in this beetle, because the differences in development of the internal reproductive organs and in the accumulation of carbohydrates and lipids both became statistically significant on the day that food consumption began to decrease. Therefore, under a temperature of 20 °C and a SD condition, the period of pre-diapause stage is the first 15 and 14 days after eclosion for female and male adults, respectively. Before the long-term storage of diapause *H. axyridis*, this period should be fully considered for diapause inducing and preparation. Sufficient food was necessary during the pre-diapause stage while less food was needed after the start of diapause. However, further research is required to determine exactly when and what nutrients should be provided to improve both survival during the long-term storage of diapause *H. axyridis* and post-diapause fitness.

## 5. Conclusions

In conclusion, we identified the period of pre-diapause stage of female and male adult *H. axyridis* and clarified key preparatory events occurring in this stage: (1) at the condition of 20 °C combined with SD condition, the first 15 and 14 days after eclosion was the pre-diapause stage of female and male adult *H. axyridis*, respectively; (2) during pre-diapause stage the development of internal reproductive organs was suppressed at early adult stage; (3) increasing of feeding amount and significant accumulation of carbohydrates and lipids were founded in pre-diapause stage. Our results not only provide a basis for further research related to the pre-diapause stage of *H. axyridis*, but also provide new clues for a better utilization of diapause strategy during mass production of this biocontrol species.
